# Complete Genome Sequence of *Paenibacillus* strain Y4.12MC10, a Novel *Paenibacillus lautus* strain Isolated from Obsidian Hot Spring in Yellowstone National Park

**DOI:** 10.4056/sigs.2605792

**Published:** 2012-07-27

**Authors:** David A. Mead, Susan Lucas, Alex Copeland, Alla Lapidus, Jan-Feng Cheng, David C. Bruce, Lynne A. Goodwin, Sam Pitluck, Olga Chertkov, Xiaojing Zhang, John C. Detter, Cliff S. Han, Roxanne Tapia, Miriam Land, Loren J. Hauser, Yun-juan Chang, Nikos C. Kyrpides, Natalia N. Ivanova, Galina Ovchinnikova, Tanja Woyke, Catherine Brumm, Rebecca Hochstein, Thomas Schoenfeld, Phillip Brumm

**Affiliations:** 1 Lucigen Corporation, Middleton, Wisconsin; 2 DOE Great Lakes Bioenergy Research Center, University of Wisconsin, Madison, Wisconsin; 3 DOE Joint Genome Institute, Walnut Creek, California; 4 Los Alamos National Laboratory, Bioscience Division, Los Alamos, New Mexico; 5 Oak Ridge National Laboratory, Oak Ridge, Tennessee

**Keywords:** *Geobacillus* sp. Y412MC10, *Paenibacillus sp.* Y412MC10, Obsidian Hot Spring

## Abstract

*Paenibacillus sp.*Y412MC10 was one of a number of organisms isolated from Obsidian Hot Spring, Yellowstone National Park, Montana, USA under permit from the National Park Service. The isolate was initially classified as a *Geobacillus sp.* Y412MC10 based on its isolation conditions and similarity to other organisms isolated from hot springs at Yellowstone National Park. Comparison of 16 S rRNA sequences within the *Bacillales* indicated that *Geobacillus sp.*Y412MC10 clustered with *Paenibacillus* species, and the organism was most closely related to *Paenibacillus lautus*. Lucigen Corp. prepared genomic DNA and the genome was sequenced, assembled, and annotated by the DOE Joint Genome Institute. The genome sequence was deposited at the NCBI in October 2009 (NC_013406). The genome of *Paenibacillus sp.* Y412MC10 consists of one circular chromosome of 7,121,665 bp with an average G+C content of 51.2%. Comparison to other *Paenibacillus* species shows the organism lacks nitrogen fixation, antibiotic production and social interaction genes reported in other paenibacilli. The Y412MC10 genome shows a high level of synteny and homology to the draft sequence of Paenibacillus sp. HGF5, an organism from the Human Microbiome Project (HMP) Reference Genomes. This, combined with genomic CAZyme analysis, suggests an intestinal, rather than environmental origin for Y412MC10.

## Introduction

Numerous novel microorganisms have been isolated from hot springs in Yellowstone National Park. Many of these organisms have been shown to possess enzymes with significant potential in biotechnological applications [1]. Among the organisms first isolated from Yellowstone hot springs are *Thermus aquaticus* [2,3], *Thermus brockianus* [4], *Acidothermus cellulollyticus* [5], and *Synechococcus* species [6]. As part of a project in conjunction with the Department of Energy Joint Genome Institute, Lucigen Corp. isolated, characterized, and sequenced a number of new isolates from Yellowstone hot springs. The bacterial isolate Y412MC10 was one of four microorganisms isolated from Obsidian Hot Spring, Yellowstone National Park, Montana, USA and submitted for whole genome sequencing. Y412MC10 was initially classified as a *Geobacillus sp.* based on its isolation conditions and morphological similarity to other organisms such as *Geobacillus* species Y412MC61 (GenBank 544556), Y412MC52 (GenBank 550542), and *Geobacillus thermoglucosidasius* C56-YS93 (GenBank 634956). The *Geobacillus sp.*Y412MC10 draft genome sequence was deposited at the NCBI in October 2009 (NC_013406) with the lineage entry indicating that it is a *Geobacillus*. Following assembly of the complete genome of Y412MC10, the 16S rRNA sequence and genome properties properly assigned the organism as a *Paenibacillus sp.* Y412MC10 represents the first *Paenibacillus sp.* isolated from a hot spring to have its genome completely sequenced.

*Paenibacillus sp.* were originally grouped in the genus *Bacillus* until 1993, when Ash *et al*. [7] proposed that members of "group 3" should be transferred to the genus *Paenibacillus*, and proposed *Paenibacillus polymyxa* as the type species. *Paenibacillus sp.* have been isolated from a wide range of environments including soil [8], the Antarctic [9], and the oral cavity of a dog [10]. *Paenibacillus sp.* are of interest for a number of reasons, including production of antibiotics [11-13], biopolymer-degrading enzymes [14-16], and their ability to fix nitrogen [17,18]. One species, *P. vortex* shows highly unusual organized growth morphologies on solid surfaces [19,20]; another species, *P. dendritiformis*, also shows unusual growth morphologies on solid surfaces [19,21,22]. Comparison of the genetic content of Y412MC10 with genomes of *Paenibacillus sp.* from other environments will give insights into the evolutionary adaptations that have occurred in the *Paenibacillus*. The organism also may be a source of novel polysaccharide degrading enzymes for use in biomass degradation.

## Classification and features

**Figure 1:**
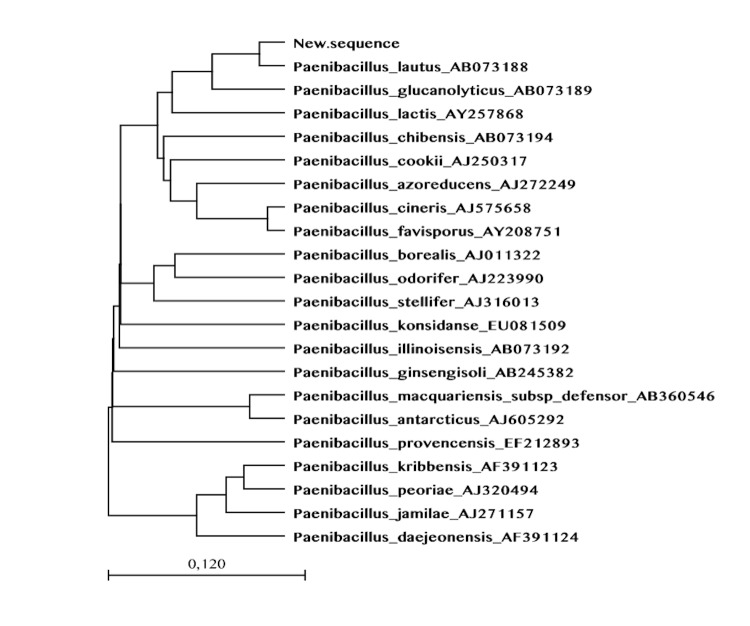
Phylogenetic tree highlighting the position of *Paenibacillus lautus* strain Y412MC10 and relative strains within the *Bacillales*. Strains used for the comparison (16S rRNA accession number) were (*Paenibacillus* Y412MC10); *Paenibacillus lautus* DSM 3035^T^ (AB073188); *Paenibacillus glucanolyticus* DSM 5162^T^ (AB073189); *Paenibacillus illinoisensis* DSM 11733^T^ (AB073192); *Paenibacillus chibensis* DSM 11731^T^ (AB073194); *Paenibacillus ginsengisoli* LMG 23406 ^T^ (AB245382); *Paenibacillus macquariensis subsp defensor* NCIB 14397 ^T^ (AB360546); *Paenibacillus kribbensis* KCTC 0766BP ^T^ (AF391123); *Paenibacillus daejeonensis* KCCM 41557 ^T^ (AF391124); *Paenibacillus borealis* DSM 13188 ^T^ (AJ011322); *Paenibacillus odorifer* DSM 15391 ^T^ (AJ223990); *Paenibacillus cookie* LMG 18419 ^T^ (AJ250317); *Paenibacillus jamilae* DSM 13815 ^T^ (AJ271157); *Paenibacillus azoreducens* DSM 13822 ^T^ (AJ272249); *Paenibacillus stellifer* DSM 14472 ^T^ (AJ316013); *Paenibacillus peoriae* DSM 8320 ^T^ (AJ320494); *Paenibacillus cineris* LMG 18349 ^T^ (AJ575658); *Paenibacillus antarcticus* LMG 22078 ^T^ (AJ605292); *Paenibacillus favisporus* LMG 20987 ^T^ (AY208751); *Paenibacillus lactis* DSM 15596 ^T^ (AY257868); *Paenibacillus provencensis* CIP 109358^T^ (EF212893); *Paenibacillus konsidanse* KCTC 13165^T^ (EU081509).

A phylogenetic tree was constructed to identify the family relationship of strain Y412MC10 (Figure 1). The tree was created using BLAST2Tree software [23]. The analysis was carried out using only type strains of validly-named organisms, and the analysis shows that Y412MC10 does not clade with known *Geobacillus* species. Rather, Y412MC10 clades in the *Paenibacillus* genus. Based on r16S analysis of validly-named organisms, Y412MC10 is most closely related to *Paenibacillus lautus* DSM 3055^T^ (AB073188). The classification of the isolate was confirmed using the EzTaxon-e server [24], again on the basis of 16S rRNA sequence data. When compared to their entire r16S database, Y412MC10 was identified as being a strain of *Paenibacillus lautus* with 99.09% identity and 100% completeness to the r16S of the type strain, *Paenibacillus lautus* NRRL NRS-666 GenBank D78472.

*Paenibacillus sp.* Y412MC10 was one of a number of organisms isolated from Obsidian Hot Spring, Yellowstone National Park, Montana, USA (44.6100594° latitude and -110.4388217° longitude) under a sampling permit from the National Park Service. The hot spring possesses a pH of 6.37 and a temperature range of 42-90°C. The organism was isolated from a sample of hot spring water by enrichment and plating on YTP-2 medium [25] (YTP-2 media contains (per liter) 2.0 g yeast extract, 2.0 g tryptone, 2.0 g sodium pyruvate, 1.0 g KCl, 2.0 g KNO3, 2.0 g Na_2_HPO_4_.7H_2_O, 0.1 g MgSO_4_, 0.03 g CaCl_2_, and 2.0 ml clarified tomato juice) at 50°C. Culture stocks were routinely maintained on YT (containing (per liter) 5.0 g yeast extract, 8.0 g tryptone, and 2.5 g NaCl) agar plates at 37°C. As part of the sequencing agreement with the Joint Genome Institute, the culture is available without restrictions from the authors. Lucigen, the National Park Service, and the Joint Genome Institute have placed no restrictions on the use of the culture or sequence data. Y412MC10 is a Gram-positive facultative anaerobe (Table 1) that grows well on a wide variety of standard lab media (YT, TB, LB). On plates, the organism grows as rods or chains of rods (Figure 2A). After growth for 6 days on plates, the cells still appear rod-shaped, but an extracellular matrix appears to surround and bind the individual cells together (light green background, Figure 2B). In liquid culture, the organism appears to also grow as a mixture of single cells and large clumps of cells surrounded by an extracellular matrix (Figure 2C). Prolonged growth on plates or in liquid culture results in sporulation of the culture; spores are subterminal with swollen sporangia.

**Table 1:** Classification and general features of *Paenibacillus* strain Y412MC10

**MIGS ID**	**Property**	**Term**	**Evidence code**
	Current classification	Domain *Bacteria*	TAS [26]
		Phylum *Firmicutes*	TAS [27-29]
		Class *Bacilli*	TAS [30,31]
		Order *Bacillales*	TAS [32,33]
		Family *Bacillaceae*	TAS [32,34]
		Genus *Paenibacillus*	TAS [35-39]
		Species *Paenibacillus lautus*	TAS [40]
		Strain Y412MC10	IDA
	Gram stain	positive	IDA
	Cell shape	rods and chains of rods	IDA
	Motility	motile	IDA
	Sporulation	sporulating	IDA
	Temperature range	mesophilic	IDA
	Optimum temperature	37°C	IDA
MIGS-22	Oxygen requirement	facultative anaerobe	IDA
	Carbon source	Carbohydrate or protein	IDA
	Energy source	chemoorganotrophic	IDA
	Electron acceptor	Oxygen, nitrate	IDA
MIGS-6	Habitat	hot spring	IDA
MIGS-6.3	Salinity	Grows in 3% NaCl	IDA
MIGS-15	Biotic relationship	free-living	IDA
MIGS-14	Pathogenicity	None, BSL1	IDA
	Isolation	Obsidian spring	IDA
MIGS-4	Geographic location	Yellowstone National Park	IDA
MIGS-5	Sample collection time	September 2003	IDA
MIGS-4.1	Latitude	44.6100594	TAS [1]
MIGS-4.2	Longitude	-110.4388217	TAS [1]
MIGS-4.3	Depth	Surface of spring	IDA
MIGS-4.4	Altitude	2416 m	TAS [1]

Evidence codes - IDA: Inferred from Direct Assay; TAS: Traceable Author Statement (i.e., a direct report exists in the literature); NAS: Non-traceable Author Statement (i.e., not directly observed for the living, isolated sample, but based on a generally accepted property for the species, or anecdotal evidence). These evidence codes are from the Gene Ontology project [41].

**Figure 2A:**
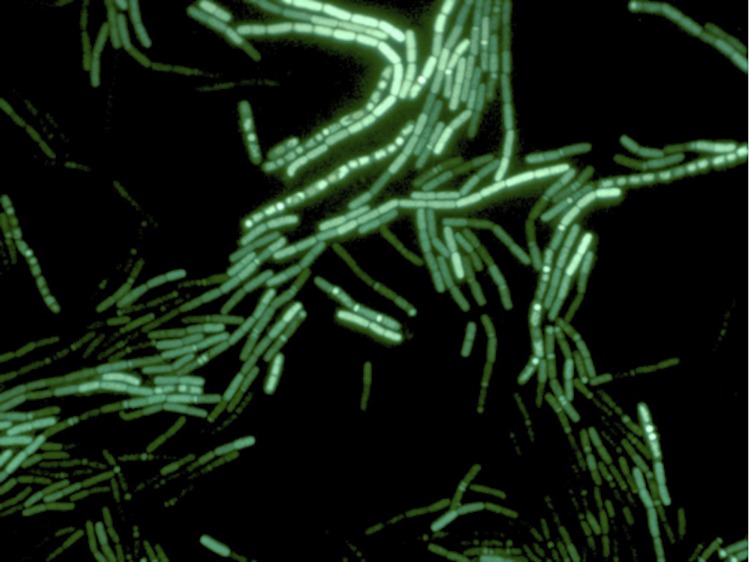
Micrograph of *Paenibacillus* strain Y412MC10 cells showing individual cells and chains of cells. Cells were streaked on YT agar and incubated 18 hr. at 37°C. A colony was removed, re-suspended in sterile water and stained using a 5 μM solution of SYTO® 9 fluorescent stain in sterile water (Molecular Probes). Dark field fluorescence microscopy was performed using a Nikon Eclipse TE2000-S epifluorescence microscope at 2000× magnification using a high-pressure Hg light source and a 500 nm emission filter.

**Figure 2B:**
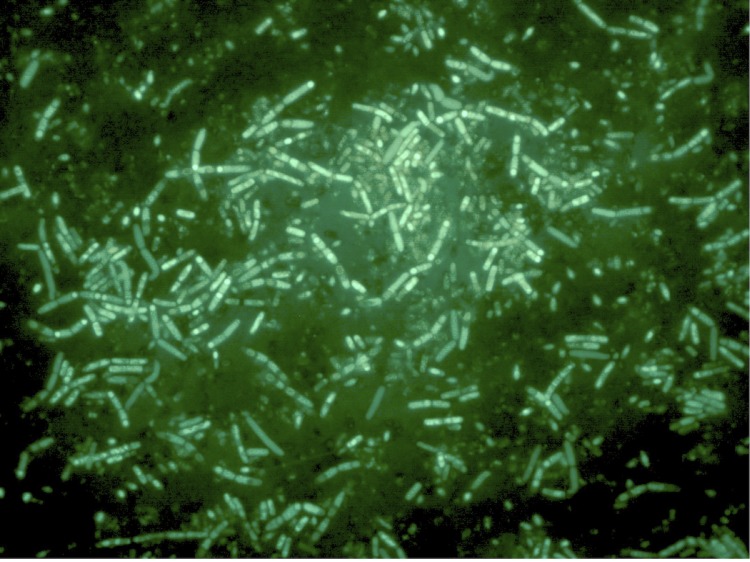
Micrograph of same plate of *Paenibacillus* strain Y412MC10 cells showing individual cells and chains of cells. Same plate as Figure 2A, but incubated 6 days at 37°C.

**Figure 2C:**
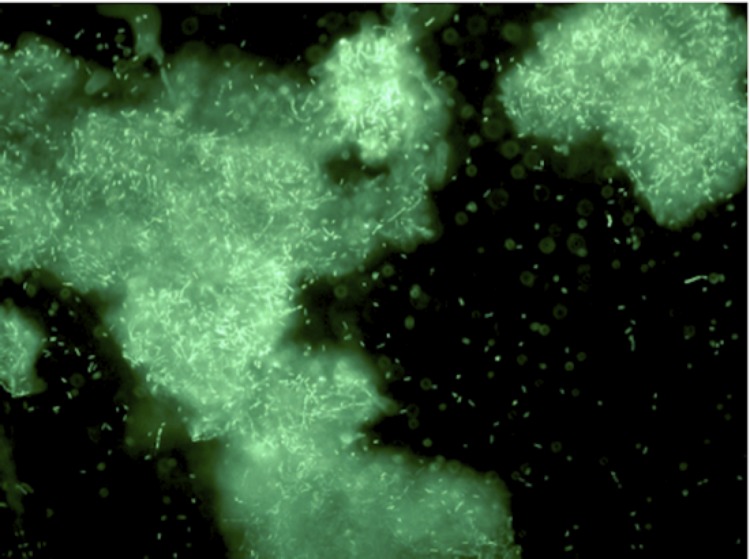
Micrograph of *Paenibacillus* strain Y412MC10 cells showing individual cells and clumps of cells. Cells were grown in YTP-2 media for 18 hours at 37°C and 200 rpm. An aliquot was removed and stained using a 5 μM solution of SYTO® 9 fluorescent stain in sterile water (Molecular Probes). Dark field fluorescence microscopy was performed using a Nikon Eclipse TE2000-S epifluorescence microscope at 200× magnification using a high-pressure Hg light source and 500 nm emission filter.

## Genome sequencing and annotation

### Genome project history

*Paenibacillus sp.*Y412MC10 was selected for sequencing on the basis of its biotechnological potential as part of the U.S. Department of Energy's Genomic Science program (formerly Genomics:GTL). The genome sequence is deposited in the Genomes On Line Database [42] (GOLD ID = Gc01127), and in GenBank (NCBI Reference Sequence = NC_013406). Sequencing, finishing and annotation were performed by the DOE Joint Genome Institute (JGI). A summary of the project information and its association with MIGS identifiers is shown in Table 2.

**Table 2:** Genome sequencing and project information

**MIGS ID**	**Property**	**Term**
MIGS-31	Finishing quality	Finished
MIGS-28	Libraries used	6 kb and 34 kb
MIGS-29	Sequencing platforms	ABI3730, 454 Titanium, Illumina GAii
MIGS-31.2	Fold coverage	5.8
MIGS-30	Assemblers	Phred/Phrap/Consed
MIGS-32	Gene calling method	Prodigal, GenePRIMP
	GenBank ID	CP001793.1
	GenBank Date of Release	October 7, 2009
	GOLD ID	Gi02010
	Project relevance	Biotechnological

### Growth conditions and DNA Isolation

For preparation of genomic DNA, liter cultures of Y412MC10 were grown from a single colony in YTP-2 medium at 37°C in flasks agitated at 200 rpm and collected by centrifugation. The cell concentrate was lysed using a combination of SDS and proteinase K, and genomic DNA was isolated using a phenol/chloroform extraction [43]. The genomic DNA was precipitated, and treated with RNase to remove residual contaminating RNA.

### Genome sequencing and assembly

The genome of *Paenibacillus lautus* Y412MC10 was sequenced at the Joint Genome Institute (JGI) [44] using Sanger sequencing with a combination of 6 kb and 34 kb DNA libraries and 454 FLX pyrosequencing done to a depth of 20× coverage [45]. Both libraries provided 5.8× coverage of the genome. Draft assemblies were based on 39,162 total reads. Solexa sequencing data was used to polish the assembly. All general aspects of library construction and sequencing performed at the JGI can be found at their website. The Phred/Phrap/Consed software package [46] was used to assemble 6-kb and fosmid libraries and to assess quality. Possible mis-assemblies were corrected; gaps between contigs were closed by 2,744 primer walks from sub-clones or 83 PCR end reads, 5 mini-libraries, and 10 PCR shatter libraries. The error rate of the completed genome sequence was 0.08, based on 49,558 total reads. Table 2 presents the project information and its association with MIGS version 2.0 compliance [47].

### Genome annotation

Genes were identified using Prodigal [48] as part of the Oak Ridge National Laboratory genome annotation pipeline, followed by a round of manual curation using the JGI GenePRIMP pipeline [49]. The predicted CDSs were translated and used to search the National Center for Biotechnology Information (NCBI) nonredundant database, UniProt, TIGRFam, Pfam, PRIAM, KEGG, COG, and InterPro databases. These data sources were combined to assert a product description for each predicted protein. Non-coding genes and miscellaneous features were predicted using tRNAscan-SE [49], RNAMMer [50], Rfam [51], TMHMM [52], and signalP [52].

## Genome properties

**Figure 3:**
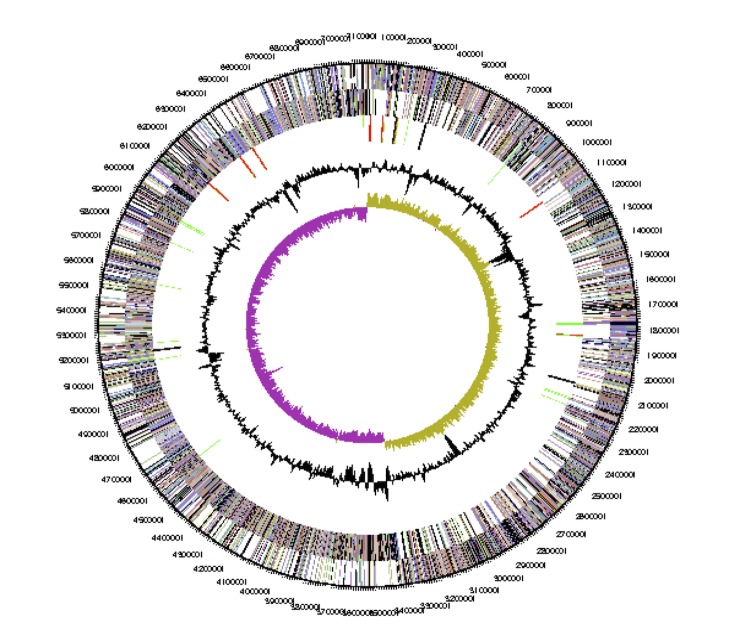
Graphical circular map of the chromosome. From outside to the center: Genes on forward strand (color by COG categories), Genes on reverse strand (color by COG categories), RNA genes (tRNAs green, rRNAs red, other RNAs black), GC content, GC skew.

The genome of *Paenibacillus lautus* Y412MC10 consists of one circular chromosome of 7,121,665 bp with an average G+C content of 51.2% (Table 3 and Figure 3). There are 73 tRNA genes, 24 rRNA genes and 4 “other” identified RNA gene. There are 6,343 predicted protein-coding regions and 105 pseudogenes in the genome. A total of 4,651 genes (72.2%) have been assigned a predicted function while the rest have been designated as hypothetical proteins. The numbers of genes assigned to each COG functional category are listed in Table 4. About 20% of the annotated genes were either not assigned to a COG or have an unknown function.

**Table 3:** Genome statistics.

**Attribute**	**Value**	**% of total^a^**
Genome size (bp)	7,121,665	100.0
DNA coding region (bp)	6,141,611	86.2
DNA G+C content (bp)	3,649,102	51.2
Number of replicons		
Total genes	6,444	100.0
RNA genes	101	1.6
rRNA operons		
Protein-coding genes	6,343	98.4
Pseudogenes		
Genes in paralog clusters	1,599	24.8
Genes assigned to COGs	4,651	72.2
Genes with signal peptides	1,506	23.4
Genes with transmembrane helices	1,921	29.8
Paralogous groups	532	

a) The total is based on either the size of the genome in base pairs or the total number of protein coding genes in the annotated genome.

**Table 4:** Number of genes associated with the 25 general COG functional categories.

**Code**	**Value**	**%age**	**Description**
J	224	3.6	Translation, ribosomal structure and biogenesis
A			RNA processing and modification
K	663	10.6	Transcription
L	152	3.2	Replication, recombination and repair
B	2	0.03	Chromatin structure and dynamics
D	213	3.4	Cell cycle control, cell division, chromosome partitioning
Y			Nuclear structure
V	244	3.9	Defense mechanisms
T	480	7.7	Signal transduction mechanisms
M	440	7.0	Cell wall/membrane/envelope biogenesis
N	130	2.1	Cell motility
Z	5	0.1	Cytoskeleton
W	0	0.0	Extracellular structures
U	41	0.7	Intracellular trafficking, secretion, and vesicular transport
O	331	5.3	Posttranslational modification, protein turnover, chaperones
C	415	6.6	Energy production and conversion
G	1,030	16.5	Carbohydrate transport and metabolism
E	788	12.6	Amino acid transport and metabolism
F	244	3.9	Nucleotide transport and metabolism
H	373	6.0	Coenzyme transport and metabolism
I	88	1.4	Lipid transport and metabolism
P	559	9.0	Inorganic ion transport and metabolism
Q	283	4.5	Secondary metabolites biosynthesis, transport and catabolism
R	872	14.0	General function prediction only
S	371	6.0	Function unknown
-	0	0.0	Not in COGs

The total is based on the total number of protein coding genes in the annotated genome. Data from [53].

## Insights from the genome sequence

Motility of *Paenibacillus* cells on solid media has been observed with a number of species. *P. lautus* is reported to spread across plates 69]. *P. vortex* shows highly unusual organized growth morphologies on solid surfaces [19,20] forming complex patterns on the plate. Another species, *P. dendritiformis*, also shows unusual growth morphologies on solid surfaces [19,21,22]. Y412MC10 was evaluated for spreading behavior on plates; the results ((Figure 4A Figure 4B Figure 4C) show definite spreading behavior for Y412MC10. The spreading behavior does not, however, appear to be as complex as reported for *P. vortex* and *P. dendritiformis.*

**Figure 4A:**
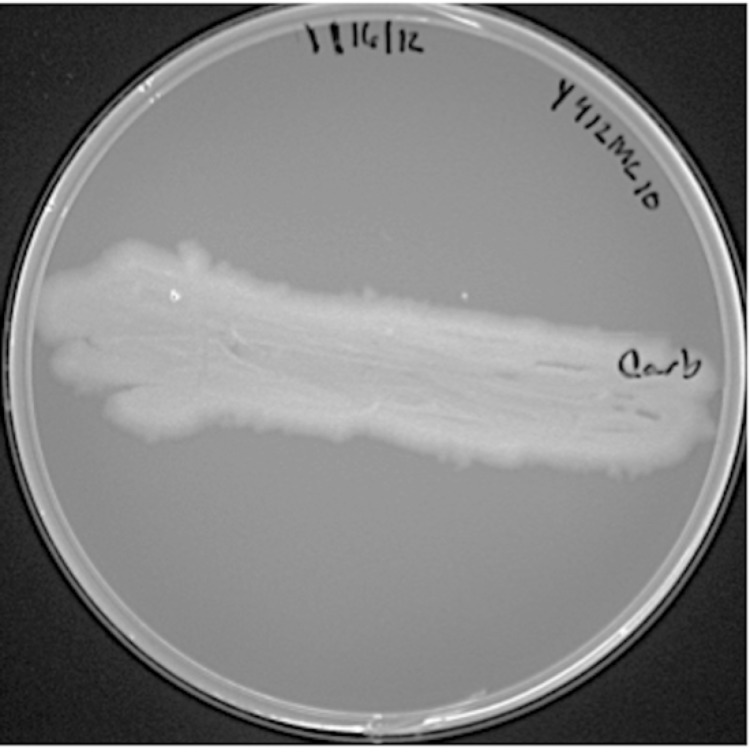
Photograph of *Paenibacillus sp.* Y412MC10 streaked on YT agar containing 100 mg/l carbenicillin and incubated at 37°C for 18 hours.

**Figure 4B:**
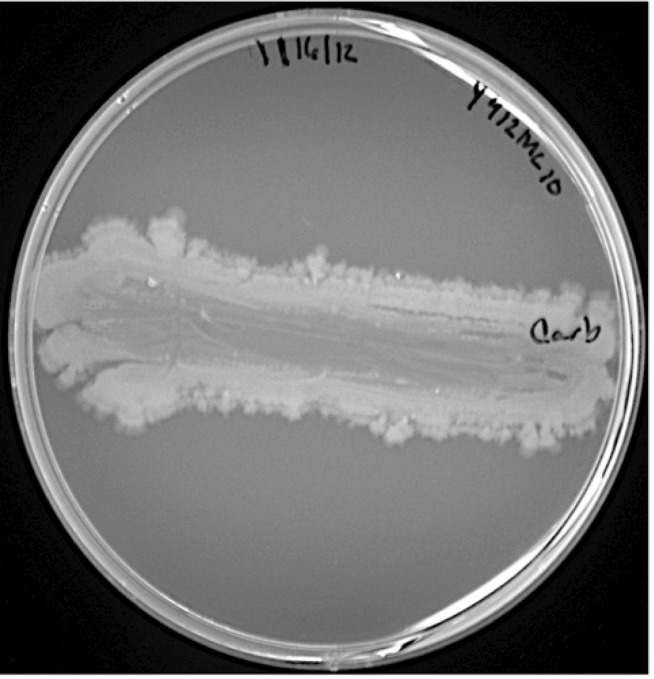
Photograph of *Paenibacillus sp.* Y412MC10 streaked on YT agar and incubated at 37°C for 50 hours. Note continued clearing of center area and significant spreading of outside edges of culture.

**Figure 4C:**
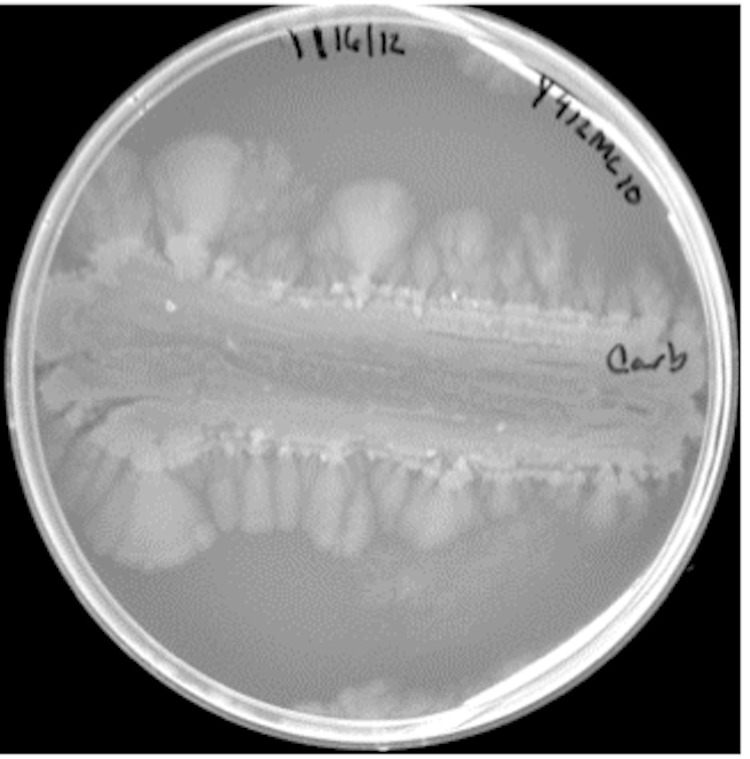
Photograph of *Paenibacillus sp.* Y412MC10 streaked on YT agar and incubated at 37°C for 168 hours. Note continued clearing of center area and significant spreading of outside edges of culture.

**Figure 5:**
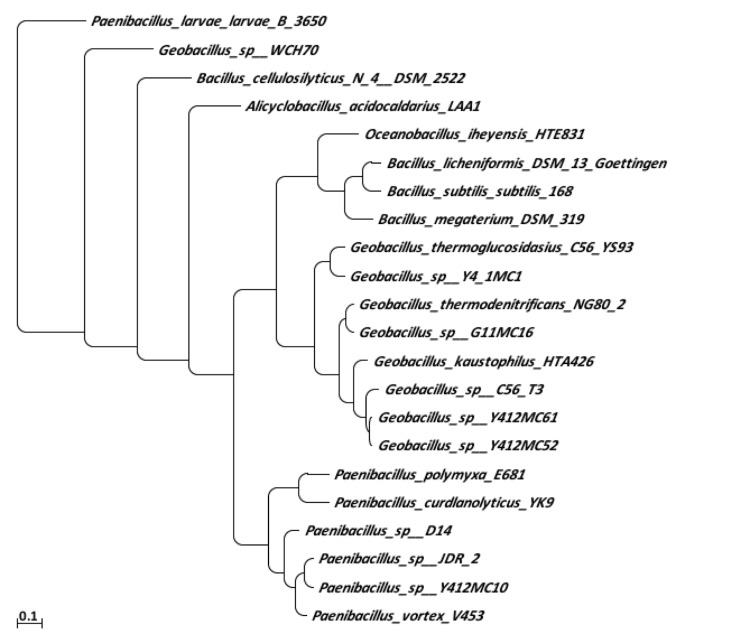
Phylogenetic tree highlighting the position of *Paenibacillus sp.* Y412MC10 and relative strains within the *Bacillales*. The strains and their corresponding GenBank taxonomy ID numbers are: *Paenibacillus sp.* JDR-2, 324057; *Paenibacillus curdlanolyticus* YK9, A717606; *Paenibacillus sp.* Y412MC10, 481743; *Paenibacillus vortex* V453, 715225; *Paenibacillus polymyxa* E681, 349520; *Paenibacillus polymyxa* SC2, 886882; *Paenibacillus mucilaginosus* KNP414, 1036673; *Paenibacillus sp.* HGF5, 908341; *Paenibacillus sp.* HGF7, 944559; *Paenibacillus larvae subsp. larvae* B-3650, 741161; *Bacillus subtilis subtilis* 168, 2243082.

To further understand the phylogenetic relationships of Y412MC10, a separate phylogenetic tree was constructed of *Paenibacillus sp.* having either complete or draft genome. This was necessary because there is little or no overlap between validly-named *Paenibacillus* strains and the *Paenibacillus* strains submitted for whole genome sequencing. The tree was created on the IMG website [54] using the alignment of 16S genes based on the SILVA database and dnadist and neighbor tools from the Phylip package [55]. *Paenibacillus sp.* JDR-2, *Paenibacillus curdlanolyticus* YK9, *Paenibacillus polymyxa* E681, *Paenibacillus polymyxa* SC2, and *Paenibacillus mucilaginosus* KNP414 are all species isolated from soil or plant roots. *Paenibacillus vortex* V453, *Paenibacillus sp.* HGF5, *Paenibacillus sp.* HGF7, are oral or intestinal human isolates. *Paenibacillus larvae subsp. larvae* B-3650, 741161 is a honey bee pathogen that attacks bee larvae. The r16S analysis (Figure 5) shows Y412MC10 is most closely related to *Paenibacillus sp.* HGF5 (NCBI Taxon ID 908341, Gold ID Gi05716), an organism being sequenced as part of the Human Microbiome Project (HMP) Reference Genomes (http://www.hmpdacc.org/reference_genomes/reference_genomes.php).

**Figure 6:**
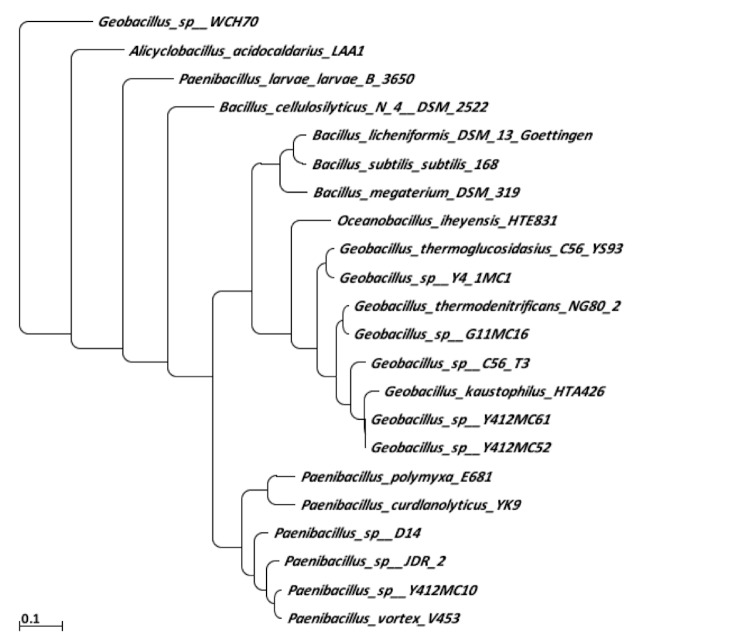
COGs whole genome comparison of selected strains. Comparison was performed as described in text; organisms and GenBank accession numbers are described in Figure 5.

**Figure 7:**
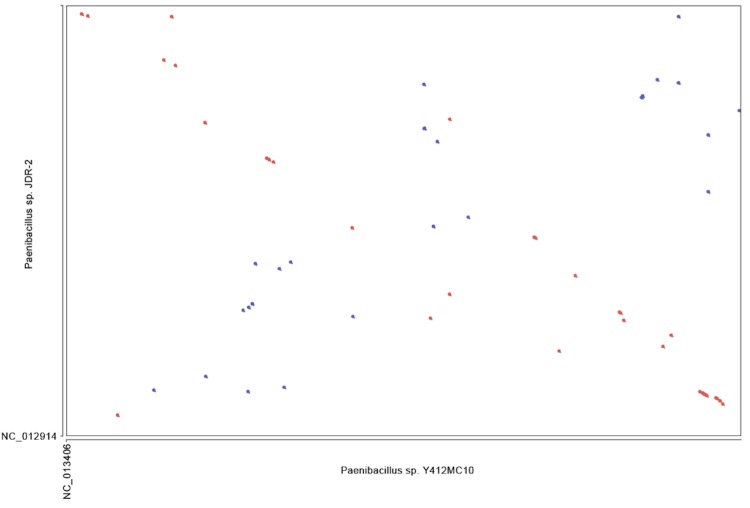
TIGRfam whole genome comparison of selected strains. Comparison was performed as described in text; organisms and GenBank accession numbers are described in Figure 5.

COG [Figure 6] and TIGRfam [Figure 7] whole genome comparisons were carried out between Y412MC10 and draft and finished genomes of closely related organisms using IMG software [56]. The results of the COGs and TIGRfam whole genome comparisons place Y412MC10 clearly among the *Paenibacillus* species, in agreement with the results from 16S analysis. The 16S analysis shows Y412MC10 is most closely related to *P. vortex* and *P. sp.* HGF5, both human isolates, and then to the two *P. polymyxa* sp. In both whole genome analyses, Y412MC10 is again most closely related to *Paenibacillus vortex* and *Paenibacillus sp.* HGF5. In the COG comparison, the other human isolate, *P. sp.* HGF7, is not closely related to *P. vortex* and *P. sp.* HGF5; in the TIGRfam comparison, HGF7 clades closely with Y412MC10, *P. vortex* and *P. sp.* HGF5. These results also suggest a mammalian, rather than environmental, ecosystem as the home of Y412MC10.

**Figure 8:**
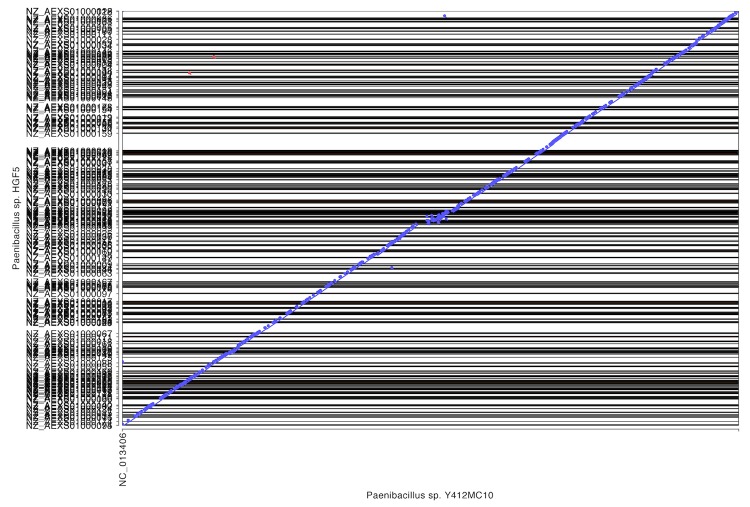
Dot plot comparison of Y412MC10 and HGF5 performed on IMG website. HGF5 draft genome contains 185 scaffolds.

**Figure 9:**
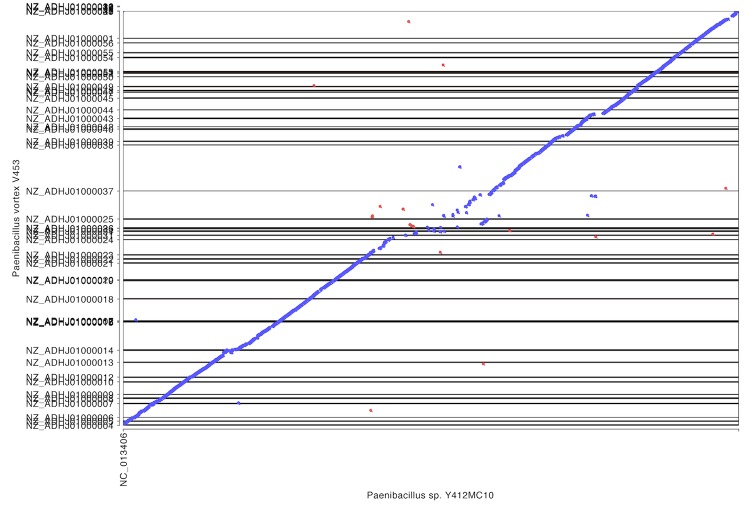
Dot plot comparison of Y412MC10 and *P. vortex* performed on IMG website. *P. vortex* draft genome contains 56 scaffolds.

To further understand the relationship between these organisms, whole genome alignments were performed using Mummer software to generate dot plot diagrams comparing pairs of genomes on the IMG website [57] using input DNA sequences directly (NUCmer). The close relationship between the genome of Y412MC10 and the genomes of HGF5 and *P. vortex* is reflected in the high levels of homology and synteny seen (Figure 8, Figure 9) with these two human isolates.

**Figure 10:**
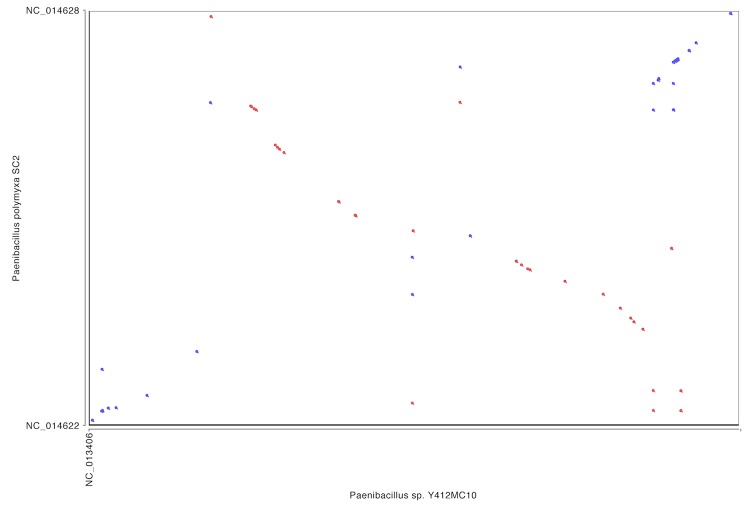
Dot plot comparison of Y412MC10 and *P. polymyxa* performed on IMG website.

**Figure 11:**
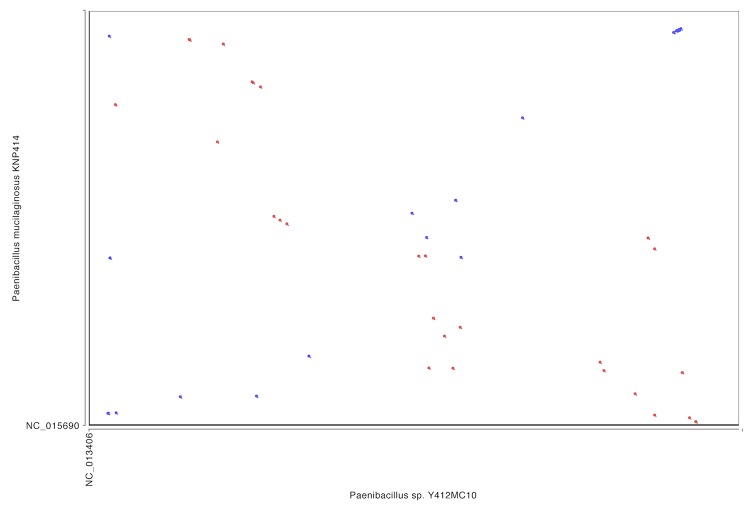
Dot plot comparison of Y412MC10 and *P mucilaginosus* performed on IMG website.

In comparison, whole genome alignment of Y412MC10 with the genomes of *P. polymyxa* and *P. mucilaginosus* show little homology or synteny between Y412MC10 and the two soil organisms (Figure 10, Figure11).

The similarity between the r16S sequences of *P. lautus* and Y412MC10 led us to examine if biochemical evidence suggested a similar habit for both. *Bacillus lautus* was first isolated from the intestinal tract of children [58]; later, the identity of the organism was re-confirmed and the organism was reclassified and renamed *Paenibacillus lautus.* Examination of the genome of Y412MC10 lends support to the hypothesis that Y412MC10 also has an intestinal origin. An analysis of the carbohydrate active enzymes (CAZY [59]) shows very low levels of GH family 5, 6, 8, 9, 10, 11, and 48 as well as no CBM 2 or 3 members, suggesting an inability to significantly degrade cellulose and hemicellulose components of biomass. CAZy analysis shows a genome enriched in GH29 and GH95 α-fucosidases; the genome is also enriched in GH38 and GH125 α-mannosidases and GH78 α-L-rhamnosidases. All these enzyme groups attack carbohydrate sidechains attached to eukaryotic glycoproteins; such glycoproteins are found in abundance in intestinal cell walls. CAZy analysis also shows a genome enriched in GH18 chitinases, GH28 polygalacturonases, GH88 unsaturated glucuronyl hydrolases, GH105 unsaturated rhamnogalacturonyl hydrolases and pectate lyase (PL) family members. These enzymes attack dietary fiber components that would be resistant to digestion by most ruminant bacteria, allowing the organism to scavenge sugars from pre-digested dietary sources. The enzymes required for bacillibactin production appear to be present in the genome of Y412MC10; bacillibactin is involved in iron acquisition. Iron is in limited supply in intestinal environments, but is present in large excess (approximately 2 μM Fe^2+^) in Obsidian hot spring. This again argues for an intestinal origin for the organism. Y4112MC10 does not possess genes usually involved in detoxification of heavy metals and sulfide found in other hot springs organisms (unpublished results). The organism also lacks antibiotic production genes, indicating it comes from an environment with excess resources, typical of the intestine. The growth temperature range and optimum of Y412MC10 is an excellent match for intestinal conditions, but a poor fit for the conditions of Obsidian hot spring, where temperatures average 79±4°C. Nitrogen fixing *Paenibacillus* have been isolated from the rhizosphere, including *Paenibacillus brasilensis* [60], and *Paenibacillus zanthoxyli* [61]. *Paenibacillus lautus* Y412MC10 has no nitrogen-fixing genes; these would be of no advantage for a free-living organism in an intestinal environment.

Complex cooperative behaviors such as those seen with *P. dendritiformis [*62*],* and *P. vortex* [19] are not observed with Y412MC10; again, these behaviors may be unnecessary for survival in the intestine. Formation of external matrices in liquid and solid cultures may be beneficial to Y412MC10 for survival; the matrix may allow attachment of the bacteria to intestinal mucosa.

## Conclusion

*Paenibacillus sp.* Y412MC10 is the first hot spring *Paenibacillus sp.* for which a whole genome sequence is available. Based on examination of the enzymes and biochemical pathways present in the organism, r16S comparison to other sequenced organisms and type strains, and whole genome comparisons, Y412MC10 appears to be of intestinal, rather than environmental origin.

 The bison herds that are present around Obsidian hot spring may be the reservoir of this organism; on multiple collection trips, bison dung was seen in and around the pool. The upper growth temperature of 50°C and/or sporulation may have contributed to Y412MC10’s survival in this otherwise inhospitable environment.

 A major need for understanding the relationships among the *Paenibacilli* is both genome sequence information on validly-named type strains and the naming of sequenced strains. The majority of sequenced strains have not been validly named, nor has significant genomic analysis been performed on type strains. The result is two, independent, phylogenetic trees that cannot be easily overlapped (compare Figure 1 and Figure 5). For both sets of data to be useful, a consensus should be reached on a system for incorporating both sets of data.
